# Focus on Clozapine Withdrawal- and Misuse-Related Cases as Reported to the European Medicines Agency (EMA) Pharmacovigilance Database

**DOI:** 10.3390/brainsci10020105

**Published:** 2020-02-16

**Authors:** Stefania Chiappini, Fabrizio Schifano, John Martin Corkery, Amira Guirguis

**Affiliations:** 1 Psychopharmacology, Drug Misuse, and Novel Psychoactive Substances Research Unit, School of Life and Medical Sciences, University of Hertfordshire, Hatfield, Hertfordshire AL10 9AB, UK; stefaniachiappini9@gmail.com (S.C.); j.corkery@herts.ac.uk (J.M.C.); 2 Swansea University Medical School, Institute of Life Sciences 2, Swansea University, Singleton Park, Swansea SA2 8PP, UK; amira.guirguis@swansea.ac.uk

**Keywords:** clozapine, adverse drug reactions, misuse, withdrawal, dependence, overdose

## Abstract

Background: Clozapine is of high clinical relevance for the management of both treatment-resistant schizophrenia and psychotic disturbances with concurrent drug misuse. Although the molecule presents with a range of well-known side-effects, its discontinuation/withdrawal syndrome has been only anecdotally described. Aims: the 2005–2018 European Medicines Agency (EMA) dataset of Adverse Drug Reactions (ADRs) was analyzed to identify and describe possible clozapine withdrawal- and misuse-/abuse-/dependence-related issues. Method: A descriptive analysis of clozapine-related ADRs was performed when available, data on ADRs’ outcome, dosage, and possible concomitant drug(s) were considered. Results: Out of 11,847 clozapine-related ADRs, some 599 (5.05%) were related to misuse/abuse/dependence/withdrawal issues, including 258 withdrawal-related (43.1%); 241 abuse-related (40.2%); and 80 intentional product misuse-related (13.3%) ADRs. A small number of overdose- and suicide-related ADRs were reported as well. Clozapine was typically (69.2%) identified alone, and most (84.7%) fatalities/high-dosage intake instances were reported in association with a history of substance abuse. Conclusions: Previous suggestions about the possibility of a clozapine discontinuation/withdrawal occurrence are here supported, but further studies are needed. However, the misuse/abuse cases here identified might be difficult to interpret, given the lack of studies highlighting the possible recreational use of clozapine. The high-dosage intake, fatal outcomes and clozapine/polydrug abuse issues reported here may, however, be a reason for concern.

## 1. Introduction

Clozapine was first synthesized in 1959 [1] and made available on the European market in 1971 for the treatment of schizophrenia [2]. If compared with remaining first-generation antipsychotic agents, clozapine is characterized by a lack of extrapyramidal side-effects [2]. After having been withdrawn from the European market in 1976 due to fatal cases of agranulocytosis, clozapine was approved by the Food and Drug Administration (FDA) in 1989 for treatment-resistant schizophrenia [2,3]. It was then approved in the USA in 2002 for reducing recurrent suicidal behavior in patients with schizophrenia or schizoaffective disorder. Other unlicensed uses include treatment-resistant bipolar disorder, aggression in patients with psychosis, and other brain disorders unresponsive to remaining treatments [4].

### 1.1. Pharmacodynamic Considerations

Clozapine is a dibenzodiazepine designated as a Multi-Acting Receptor-Targeted Antipsychotic (MARTA), deriving its therapeutic effects from its action across various neurotransmitter systems [4,5]. In fact, a combination of pharmacological effects is unique to clozapine: (1) although the molecule presents with affinity levels for a range of dopamine/DA receptors (e.g., D1, D2, D3, D4, and D5; [6]), the blockade of dopamine 2 and 4 receptors is particularly relevant, with a preferential affinity for D4 over D2 receptors; this blockade seems to be effective in reducing positive symptoms of psychosis and stabilizing affective symptoms [4]; (2) 5-hydroxytryptamine/5-HT 2A receptor antagonism, causing enhancement of DA release in certain brain regions, and thus reducing motor side-effects and possibly improving cognitive and affective symptoms associated with schizophrenia [4]. Conversely, the clinical effects of the reported interactions at 5HT2C; 5HT1A; 5-HT6; and 5-HT7 receptors [6] might be less clear; (3) α2C-adrenergic receptor blockade; this may contribute to the clozapine-related improvement of cognitive function [7]; (4) significant antimuscarinic and antihistaminergic H1 effects, which may well contribute to the central effect [8]; and (5) possible modulatory actions on a dysfunctional glutamatergic system, improving schizophrenia symptoms and contrasting illness progression [1,4,9,10,11]. More precisely, clozapine interacts with the N-methyl-D-aspartate (NMDA) receptor complex, affecting either the glycine site of the NMDA receptor or tentatively inhibiting the glycine transporter [12]. Moreover, both clozapine and its major metabolite N-desmethylclozapine behave as: (i) delta-opioid receptor agonists [13,14,15,16,17]; (ii) cannabinoid CB1 receptor agonists [18]; and (iii) antagonists at muscarinic receptors [8]. Indeed, these i-iii pharmacological activities are, per se, typically associated with the occurrence of pleasurable effects [19,20,21,22], which could suggest a theoretical potential for clozapine to be misused by vulnerable individuals. Finally, further pharmacological clozapine activities which should be better understood in terms of their clinical effects may include both interaction with GABA-B receptors [22]; and antagonistic actions at D (2)/D (3)/D (4) receptors [23].

The therapeutic response depends on plasma clozapine concentrations, which may be influenced by many factors such as age, gender, and smoking [5], and should normally reach 350 ng/mL [4,5]; levels greater than 700 ng/mL are often not well tolerated [4]. Clozapine is metabolized by CYP450 enzymes, specifically by CYP450 1A2, 2D6 and 3A4 enzymes [5], and its metabolism may be influenced by CYP450 1A2 inhibitors, such as fluvoxamine and ciprofloxacin [14]. Remaining interactions may occur with both strong CYP450 2D6 inhibitors (e.g., bupropion, duloxetine, paroxetine, fluoxetine) and strong CYP450 3A4 (e.g., ketoconazole) inhibitors [4,15].

### 1.2. Clozapine Abuse Issues and Substance Use Disorders

Substance use disorders (SUDs), typically involving alcohol, cannabis, and cocaine, commonly occur in patients with schizophrenia, supposedly due to epidemiological and genetic determinants of risk for both psychosis and addiction [1,16]. This co-occurrence (‘dual diagnosis’) has a negative effect on the course of schizophrenia, due to increased rates of hospitalization, decreased compliance with medication, increased violence and suicide, general deterioration of the patients’ condition, and overall increased societal costs [1,17]. In particular, the positive symptoms of schizophrenia are generally exacerbated by the intake of stimulant drugs, such as cocaine, amphetamine derivatives [24], and synthetic cathinones [25]. Due to clozapine’s effectiveness, there has been support for considering the molecule in limiting substance use in patients with schizophrenia [26], to achieve both a reduction in substance use [27,28] and an improvement in positive/negative schizophrenia symptoms [29]. Additionally, differently from other antipsychotics, such as quetiapine, which has showed in recent years to be a strong potential for misuse and abuse [30,31,32,33], the recreational use of clozapine has not been noted in the literature. Conversely, clozapine withdrawal is a phenomenon which has already been described, even at therapeutic dosages [9]. However, the risk of withdrawal may arguably be significant only when a psychoactive molecule is being misused/ingested at higher dosages. Since SUD patients may be vulnerable to misuse prescribed medications, one could argue that it is relevant to identify and assess any possible clozapine misuse/abuse/withdrawal and dependence issues.

### 1.3. Aims

We aimed here at identifying and describing the number of European Medicines Agency (EMA) database cases of misuse, abuse, dependence, and withdrawal specifically relating to clozapine; suicide-related cases and fatalities were also considered.

## 2. Methods

### 2.1. Data Source and Acquisition

EudraVigilance (EV) manages and analyzes information on suspected Adverse Drug Reactions (ADRs) to medicines that have been authorized in the European Economic Area (EEA), according to Directive 2001/83/EC and Regulation (EC) No 726/2004 [34]. In order to investigate the aspects above, EV was specifically requested to provide all Level 2A data [34] comprising case reports of clozapine-related misuse, abuse, dependence, and withdrawal ADRs, which were obtained in the form of line listings, relating to the 2005–June 2018 time-frame. Differently from the publicly available data from the EV website, Level 2A data were presented as Excel sheets divided into information sections reporting in a standardized format according to the Medical Dictionary for Regulatory Activities (MedDRA), which is the internationally agreed list of terms that supports the coding of ADRs [35], which are identified through Preferred Terms (PT). Indeed, MedDRA is a comprehensive and highly standardized medical terminology developed by the International Council for Harmonization of Technical Requirements for Pharmaceuticals for Human Use (ICH) (https://www.meddra.org/) in the late 1990s to facilitate the international sharing of regulatory information for medical products. Such listings showed all information related to the ADR, the patient, the drug, the reporter, and the diagnosis. In line with previous studies from our group [36,37,38,39], the ADRs considered here were, per se, voluntary and unsolicited communications [34] reported by both Regulatory Authorities of the EU Member States where the reaction occurred and/or by the Marketing Authorization Holders for those ADRs occurring outside the EEA. Individual Case Safety Reports, such as voluntary reports, refer to the format and content for the reporting of one or several suspected ADRs in relation to a medicinal product that occur in a single patient at a specific point of time [40]. ‘Suspect’ ADRs were selected, meaning clozapine was considered as ‘suspected’ for the reaction reported [34]. Within the standardized MedDRA Query (SMQ) ‘drug abuse, dependence and withdrawal’ section, we identified the following ADRs: dependence, drug abuse, drug abuser, drug dependence, drug diversion, drug withdrawal convulsions, drug withdrawal syndrome, drug withdrawal neonatal syndrome, intentional product misuse, product use issue, substance abuse, and withdrawal syndrome. For the definition of ‘abuse’; ‘addiction’; ‘dependence’; and ‘misuse’ please refer to references [35,36,37,38,39,40]. Moreover, in accordance with MedDRA, ‘withdrawal’ is identified in association with the abrupt cessation or reduction in intake of a drug in a habituated person. A substance-specific syndrome may follow, with withdrawal symptoms varying according to the psychoactive substance used and generally opposite the acute effects of drug. These include nonspecific symptoms e.g., nausea, diarrhea or constipation, profuse sweating, increase in respiratory rate, and tachycardia; remaining common symptoms include anxiety, restlessness, irritability, insomnia, and impaired attention [35].

### 2.2. Data Analysis

Each case report may refer to one or more reporter; one or more ADR(s); as well as to one or more medicinal product(s). Therefore, a case may be represented by more than one row in the other line listings. All rows of the case have the same ‘EV Local Report Number’, unequivocally identifying an individual case. Thus, the number of suspected ADRs can be different from the number of case reports as one case report may refer to several suspected ADRs. Moreover, the number of patients can be different from the number of case reports as a patient may have been described in more than one case. Finally, ADRs’ numbers differed from those referring to case reports/single patients since different reporters/senders could have independently flagged the same ADR to the EMA.

Patients’ data were analyzed using a range of parameters, including: socio-demographic characteristics (age and gender); source/reporter country (EEA or non-EEA) and reporter qualification (i.e., pharmacist, physician); outcomes (fatal, recovered, resolved); clozapine dosage; possible concomitant drug(s); and diagnosis/reporter’s comments, if recorded [37]. The analysis included cases of overdoses, suicides, and fatalities.

Suicidal behavior was here defined as ‘completed suicide’, ‘intentional self-injury’, ‘suicidal behavior’, ‘suicidal ideation’, ‘suicide attempt’, ‘self-injurious ideation’, and ‘intentional self-injury.’ ‘Overdose’, including ‘intentional overdose’, was not necessarily interpreted as being a suicidal attempt [35]. The analysis included here both a descriptive study of the dataset and the Confidence Interval (CI) values [41].

### 2.3. Ethics’ Issues

Complying with applicable Personal Data Protection legislation (Regulation (EC) No 45/2001 and Regulation (EC) No 1049/2001 on the protection of privacy and integrity of individuals, certain data elements, including names/identifiers of individuals involved or country-specific information were not disclosed by the EMA to safeguard the identity of individuals [42]. The study was ethically approved in March 2018 by the University of Hertfordshire Ethics’ Committee, with reference number LMS/PGR/UH/03234.

## 3. Results

The EMA dataset included a total of 13,596 clozapine-related ADRs. A total of 11,847 ADRs were considered as ‘suspect’, i.e., as suspected to be related with clozapine. Of those, 599/11,847 (5.05% CI 95% 597–601) related to misuse/abuse/dependence/withdrawal ADRs and were associated with 559 unique subjects. These included: 258 ADRs (258/599 = 43.1% CI 95% 256–260) relating to ‘withdrawal syndrome’ issues; 241 abuse-related ADRs (241/599 = 40.2% CI 95% 239–242); and 80 ADRs (80/599 = 13.3% CI 95% 76–84) relating to ‘intentional product misuse.’ A small number of overdose- (*n* = 29) and suicidal behavior-related ADRs (*n* = 29) were reported here, resulting in death, respectively, in two and 11 cases (Table 1).

Patients were typically males (379/559 = 67.8% CI 95% 378–380), in the 18–65 years age range. Most typical senders were pharmaceutical companies (303/559 = 54.2% CI 95% 301.5–304.5) and regulatory authorities (241/559 = 43.11% CI 95% 239–243) (Table 2).

**Figure 1:**
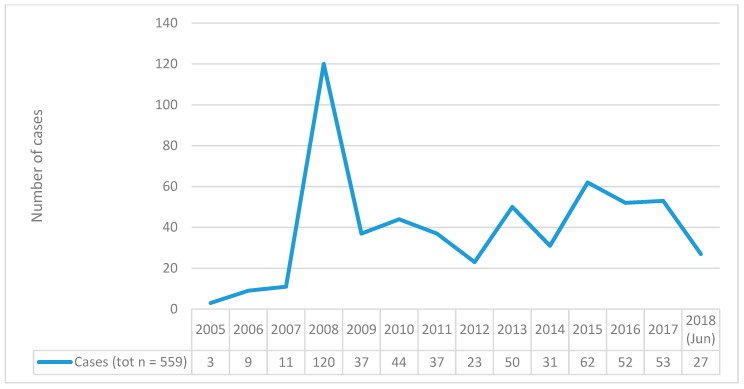
Total Number of clozapine-related misuse, abuse, dependence, withdrawal cases reported to EudraVigilance (EV) per year (2005–June 2018).

The number of cases increased year by year, with a peak in 2008 (120/559 cases) (Figure 1).

**Figure 2:**
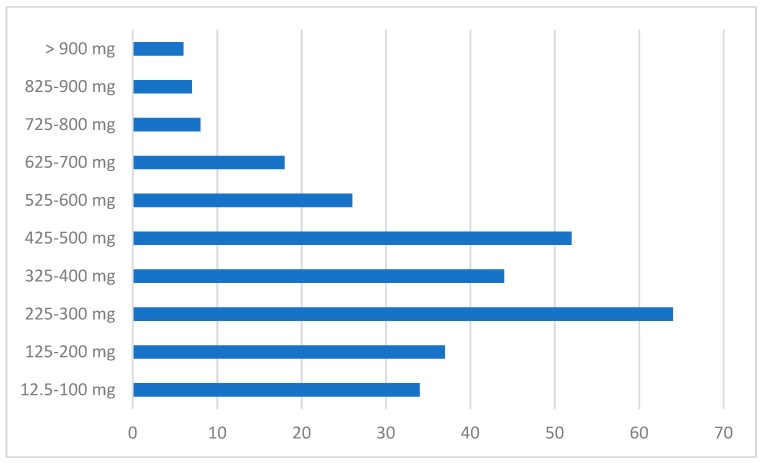
Dosages of clozapine reported to the EudraVigilance (EV) dataset of clozapine-related misuse, abuse, dependence, withdrawal ADRs.

Oral intake occurred here in 533/559 cases (95.3% CI 95% 532.5–533.5); when recorded, clozapine dosages varied from 12.5 mg/day to high/unlicensed levels (i.e., 2800–5600 mg/day; Figure 2). Only a few cases (*n* = 7), however, reported high (e.g., >1000 mg) levels. When the relating clinical data were made available, these cases were typically described as ‘intentional self-injury’, ‘completed suicide’, and ‘drug abuse’).

Clozapine was the sole molecule reported in 387/559 (69.2% of cases; CI 95% 386–388); remaining drugs included: first/second generation antipsychotics (55/559 cases; 9.8%); benzodiazepines (clonazepam, diazepam, and lorazepam; 54/559 cases; 9.7%); antidepressants (citalopram, escitalopram, sertraline, and venlafaxine; 33/559 cases; 5.9%); and mood stabilizers (valproic acid, carbamazepine, lamotrigine, lithium, and gabapentin; 20/559 cases; 3.6%). Illicit drugs most typically reported here were opioids (15/559 cases), amphetamines (10/559), cannabis, and alcohol (each in seven cases). In 30 cases, two or more prescribed/recreational drugs were recorded in association with clozapine (Table 3).

The ADRs required in most cases a prolonged hospitalization (298/559 = 53.3% CI 95% 296.5–299.5), while some 46 cases (46/559 = 8.2%) resulted in death (Table 2 and Table 4). Of these 46 clozapine-related fatalities, 39 (84.7%) were associated with a previous history of substance abuse (Table 4).

In a number of cases, reporters had omitted some relevant details, including diagnosis, drugs involved, and, more importantly, both the white blood cell (WBC) and the absolute neutrophil count (ANC) results. Out of a total of 599 ADRs, referring to 559 individual cases, some 149 ADRs (42 individual cases) mentioned that the WBC count had been carried out. However, the results were reported in only 123 ADRs (32 individual cases). For 68 of these ADRs, the WBC/ANC count was within the normal range; while 42 and 13 ADRs were, respectively, associated with either leukopenia or increased WBC count levels. However, since modifications overtime of WBC count levels were not reported, it would be problematic to unequivocally conclude from here if clozapine had been stopped in association with the occurrence of WBC count abnormalities.

A total of 28 cases reported here catatonia, six of which were specifically described as being associated with clozapine withdrawal issues; further reporters’ comments relating to overdose, suicidal behavior, withdrawal, and abuse issues are listed in Table 5.

## 4. Discussion

To the best of our knowledge, this is the largest collection of literature data relating to clozapine withdrawal and misuse/abuse/dependence cases. Our analysis found 599 ADRs of interest (relating to 559 individuals), representing 5.05% of all 11,847 reports submitted to the EMA EV between 2005 and 2018 and judged as ‘suspect.’ In contrast with the related knowledge on this topic, mostly focused on small case reports/series [10,43,44,45,46,47,48], current findings referred to high numbers of patients presenting with clozapine withdrawal and misusing issues.

Clozapine ADRs of interest showed a peak in 2008, but an overall increase was observed between 2010 and 2018. One could argue that this upward trend may have been associated with a range of factors, including the increasing rates of worldwide availability of clozapine due to an overwhelming evidence of its effectiveness [2,49,50,51] and/or a major awareness of the relevant and significant impact of pharmacovigilance processes and issues, resulting in a rise of spontaneous reporting practices related to safety monitoring of medicinal products in general [52].

Withdrawal cases collected here were recorded according to the related PTs defined in the MedDRA dictionary [35], where withdrawal is described as “the abrupt cessation of a drug use in a habituated person; and a substance specific syndrome following cessation or reduction in intake of a psychoactive substance previously used regularly”. Clozapine withdrawal/discontinuation ADRs were the most frequently reported and, as such, current findings confirmed and expanded on previous anecdotal data [6,53,54]. In association with a sudden discontinuation of clozapine, which may be required in cases of blood dyscrasia or suspected myocarditis, instances of withdrawal have already been reported [6,44,54,55]. It is of clear interest that a case of clozapine withdrawal neonatal syndrome was specifically mentioned here as well. The clozapine multi-receptor agonism/antagonism is likely responsible for the occurrence of discontinuation/withdrawal symptoms. Indeed, the clozapine pharmacodynamic profile may well include: (a) a dopaminergic super-sensitivity, with the risk of a dopaminergic psychosis and symptoms such as dystonias, dyskinesias, and catatonia [38,56,57,58,59]; (b) a cholinergic rebound, inducing in vulnerable patients a rapid worsening of psychosis, agitation, confusion, insomnia, and symptoms including nausea, vomiting, diarrhea, headache, diaphoresis, and abnormal movements, such as dystonias and dyskinesias [6,54,56,57,60,61,62,63,64]. Consistent with this, symptoms appear to regress rapidly with the help of anti-cholinergic drugs; (c) a serotonergic syndrome, which may occur even without the concomitant use of a serotonergic agent [10,39,61]. In fact, acting as a 5-HT2A antagonist, long-term clozapine use may be associated with receptor downregulation, and thus, its abrupt discontinuation might lead to receptors’ upregulation [65,66]; (d) a sudden decrease in gamma-aminobutyric acid (GABA) activity, with the development of catatonic symptoms which may include, mutism, waxy flexibility, staring, posturing, mannerisms, negativism, and also restless, irrelevant speech, and psychomotor agitation [6,67]. The clozapine agonist action on GABA receptors can explain both the drug-drug interaction between clozapine and benzodiazepines, and the flumazenil therapeutic effect in clozapine intoxication cases [6]; and (e) a modification of norepinephrine levels, with clozapine abrupt discontinuation in chronic patients possibly resulting in an increase in suicidal behavior [1,68]. As it may occur with remaining psychotropics, such as antidepressants [69,70], the existence of a discontinuation syndrome following an abrupt stoppage of, or marked reduction in, the dosage of a drug taken on a regular basis does not necessarily mean that a drug causes dependence. Discontinuation should be seen here as distinct from the withdrawal scenario associated with alcohol and other addictive substances, a scenario which commonly presents together with craving, drug seeking behavior, and the inability to stop drug use [71,72]. Thus, if a discontinuation of clozapine is needed, the molecule should be gradually tapered off over several weeks rather than abruptly discontinued, except in cases of emergency (e.g., agranulocytosis), and only with close clinical monitoring [10,67,73]. There are no established guidelines regarding which antipsychotic to choose after withdrawal of clozapine [73], although anticholinergics and olanzapine may be the treatment of choice for preventing withdrawal [53].

The reporters’ narrative here formally submitted in a few cases was consistent with both drug seeking and diversion behavior (e.g., “…clozapine as a drug of abuse…”; and “…patient possibly has used Leponex as drug of abuse or he bought Leponex for drug abuse by others…”.). Considering the current misuse/abuse issues, the number of clozapine related ADRs (e.g., 326 ADRs; referring to: ‘drug abuse’, ‘drug abuser’, ‘drug diversion’, ‘intentional product misuse’, ‘product use issue’, and ‘substance abuse’) here identified may be difficult to interpret. In fact, in comparing quetiapine- and other second-generation antipsychotic-related intentional abuse exposures reported to the US National Poison Data System, Klein et al. [74] suggested that clozapine and olanzapine were significantly associated with frequent instances of severe central effects, including: lethargy/drowsiness/slurred speech; agitation/irritability; confusion and hallucinations. However, no use of clozapine as a recreational drug was reported. One could argue that at least a proportion of these ADRs involved subjects suffering from both schizophrenia and a co-occurring SUD. Furthermore, current findings here did not identify any idiosyncratic intake modalities (e.g., intravenous use) that are typical of a substance misuse behavior. Hypothetically, putative levels of clozapine misuse liability might be tentatively explained considering the range of its pharmacodynamics activities, and the occurrence of rewarding and pleasurable effects due to the agonism at both delta-opioid [13,17,75] and cannabinoid CB1 receptors [18]; and the antagonism at muscarinic receptors [76,77]. Additionally, although clozapine was here ingested on its own in some 69% of cases, remaining antipsychotics and benzodiazepines were the drugs most frequently reported in association. Polypharmacy ingestion may have facilitated the occurrence of synergistic reactions, and hence the EMA ADRs’ reporting, due to possible increase in clozapine plasma concentrations associated with metabolism inhibition [14]. Relatively small recreational drugs’ numbers were identified here as well, and this may have been associated with complex pharmacological interactions. Indeed, clozapine pre-treatment may increase cocaine concentrations, but significantly reduce subjective responses to cocaine [78].

Regarding the ADRs’ outcomes, present figures seem to be a reason for concern, since most cases (298/559 = 53.3%) required a prolonged hospitalization. Furthermore, some 46 (8.2% of 559 subjects) fatalities were here reported, and these mostly occurred in the context of: high dosage clozapine intake; suicidal behavior; and/or polydrug abuse. Although the EMA suggests a maximum clozapine dosage of up to 900 mg/day and warns about side-effects occurring at doses over 450 mg/day [79], cases reported here were at times associated with massive dosages (e.g., in the range of 2800–5600 mg), although most of these cases were associated with overdose/suicide instances. These rates are a reason for concern but seem to be consistent with the suggestion that those subjects diagnosed with treatment-resistant schizophrenia, and hence, who are typically being prescribed with clozapine, present with a higher risk of attempted/completed suicide in comparison to the general population [80]. Clozapine overdose may be lethal due to changes in heart rhythm, respiratory depression, and altered state of consciousness [4,81,82]; clozapine high-dosage ingestion may be associated with a mortality rate of approximately 12% [83], with fatalities most typically being associated with cardiac insufficiency and aspiration pneumonia observed with dosages higher than 2 g [45,46,47,48]. Although cases of full recovery after ingestion of either high dosages [46] and/or associated with very high plasma levels (>9000 ng/mL) [45] have been reported, ingestion of 400 mg in a patient not previously treated may be life-threatening [46].

### 4.1. Limitations

Overall, pharmacovigilance systems such as the EV in Europe, the Medicines and Healthcare products Regulatory Agency (MHRA) in the United Kingdom and the FDA Adverse Event Reporting System (FAERS) are considered important because the analysis of suspected ADRs may deal with early detection of possible drug safety signals [34]. However, the study of ADRs alone may not be enough to prove causality between a certain suspected reaction and a specific medicine. Any case report should be considered and assessed together with all available data, including case reports worldwide, clinical trials, epidemiological studies, and toxicological investigations.

Unfortunately, the dataset did not typically report here any clinical data, including information on the current/past psychopathology and the medications that were prescribed in association. Furthermore, the study of ADRs may be influenced by the molecule’s availability and extent of use, although precise data on the worldwide prescription rates for clozapine were not here identified or available. Moreover, in being voluntarily reported/submitted, the number of ADRs could have been conditioned by both the nature of the reaction and the public awareness of a safety concern, with underreporting possibly having here occurred. Additionally, some data could have been unavailable, invalid or redacted.

### 4.2. Conclusions

Current findings focused on a range of clozapine withdrawal and misuse/abuse/dependence issues. It was here confirmed, on a large-scale basis, that, following the abrupt reduction of clozapine dosage a discontinuation/withdrawal syndrome may occur. This may involve possible severe and long-lasting symptoms [10,59,67,84]. Moreover, although this may be an unusual event, it is here suggested that cases of misuse/abuse/dependence could occur in patients with a concomitant SUD and this is despite clozapine being of clinical value for the treatment of dual diagnosis. Conversely, current findings emphasized a range of severe and fatal health consequences associated with clozapine high-dosage intake, and patients taking clozapine should be educated thoroughly about the risks associated with polydrug intake. In being a prescribed drug of highly significant clinical value [85], increased awareness is essential towards prevention, diagnosis, and treatment of cases of clozapine abuse/recreational use in patients diagnosed with schizophrenia [86].

## Figures and Tables

**Table 1:** Analysis of the EudraVigilance (EV) clozapine-related misuse/abuse/dependence and withdrawal ADRs (2005–June 2018).

EudraVigilance (EV) Clozapine-Related Misuse/Abuse/Dependence and Withdrawal ADRs (2005–June 2018)	*n*
Total “suspect” clozapine-related ADRs	11,847
Clozapine-related ‘abuse, dependence and withdrawal’ ADRs	599 (*CI* 95% 595–603) (*n* individual cases = 559)
Drug abuse	198
Drug abuser	1
Substance abuse	42
Dependence	7
Drug dependence	6
Drug diversion	1
Intentional product misuse	80
Product use issue	4
Drug withdrawal convulsions	1
Drug withdrawal neonatal syndrome	1
Drug withdrawal syndrome	91
Withdrawal syndrome	165
Further issues emerging from the analysis of clozapine ADRs’ dataset	
Intentional overdose	12
Overdose	17
Completed suicide	9
Intentional self-injury	4
Suicidal behavior	1
Suicidal ideation	4
Suicide attempt	7
Self-injurious ideation	4

**Table 2:** Description of clozapine-related misuse, abuse, dependence and withdrawal cases. F: female; M: male; N: no; Y: yes.

Clozapine-Related Misuse, Abuse, Dependence and Withdrawal Cases	*n* of Unique Patients
	559
**Age range (years)**	3 neonates, 1 child (5 years), 2 adolescents (15–16 years), 78 adults (18–65 years), 1 elderly (67 years), 474 Not specified
**Gender**	171 F, 379 M, 9 Not Specified
**Sender**	241 Regulatory authority, 303 Pharmaceutical company, 9 Other (distributor, study sponsor, contract research organization), 6 Not Specified
**Outcome**	
Resulted in death	46 Y, 448 N, 65 Not Specified
Life threatening	35 Y, 447 N, 77 Not Specified
Required a prolonged hospitalization	298 Y, 219 N, 42 Not Specified
Disabling	8 Y, 467 N, 84 Not Specified

**Table 3:** Clozapine alone or in combination rates of identification, as recorded by the EudraVigilance (EV) dataset of misuse, abuse, dependence, and withdrawal cases.

Clozapine-Related Misuse, Abuse, Dependence and Withdrawal Cases Recorded by the EudraVigilance (EV) Dataset	Tot (*n* = 559)
Clozapine ingested as a lone drug	387 (69.2%)
Clozapine identified in combination with remaining drugs	172 (30.7%)
**Prescribing drugs**	
Other antipsychotics	55
Benzodiazepines	54
Antidepressants	33
Mood stabilizers	20
Z-drugs	4
**Recreational drugs**	
Opioids	15
Amphetamine derivatives	10
Cannabis	7
Alcohol	7
Cocaine	4
Ketamine	1

**Table 4:** Description of the clozapine-related fatalities’ cases reported to the EudraVigilance (EV) dataset (2005–June 2018). F: female; M: male.

		Clozapine Dosage (mg)	Concomitant Drugs	Medical History	Reactions according to the MedDRA Dictionary (Preferred Terms-PT)
1	M, Adult (30 yy)	200 mg	Benzodiazepines Alcohol		Intentional product misuse; Antipsychotic drug level increased (clozapine plasmatic 1.1 mg/mL); Blood pressure decreased; Loss of consciousness; Poisoning; Dysarthria; Loss of consciousness; Blood alcohol increased
2	M, Adult (48 yy)	N/A	Cocaine Opioids Z-drugs	Hypertension; Schizophrenia; Chronic obstructive pulmonary disease; Arteriosclerosis	Substance abuse; Toxicity to various agents
3	M, Adult (25 yy)	300 mg	Benzodiazepines Opioids		Cardiac arrest; Arrhythmia Toxicity to various agents (methadone and clozapine); Drug abuse; Drug level increased (methadone and clozapine)
4	M, Adult (44 yy)	N/A	Opioids	Hypertension; Alcoholism; Depression; Epigastric discomfort; Anxiety	Pulmonary embolism; Drug abuse
5	M, Adult	400 mg	Alcohol		Liver function test abnormal; Blood alkaline phosphatase increased; Gamma-glutamyl transferase increased; Asphyxia; Alcohol abuse; Drug abuse (heroin)
6	M, Adult (24yy)	N/A	Alcohol	Anemia	Drug abuse (Clozaril abuse)
7	F	300 mg		Alcoholism; Mental impairment; Depression; Treatment noncompliance; Deep vein thrombosis	Death; Withdrawal syndrome; Acute psychosis; Cognitive disorder; Amnesia; Speech disorder; Gait disturbance; Chills
8	F, Adult (54yy)	350 mg	Benzodiazepines		Withdrawal syndrome; Insomnia; Completed suicide
9	M, Adult (66 yy)	N/A			Completed suicide; Drug abuse
10	F, Adult (25yy)	N/A	Antidepressants		Completed suicide; Drug abuse
11.	M, Adult (31yy)	N/A			Completed suicide; Drug abuse
12	M	N/A		Obesity; Gastroesophageal reflux; Drug abuse	Drug abuse; Death
13	F	N/A	Cocaine Benzodiazepines Opioids	Drug dependence	Drug abuse (cocaine, leponex and probably benzodiazepines); Death; Hyperthermia malignant; Cardiac arrest; Circulatory collapse; Delirium
14	M	N/A		Hepatitis	Drug abuse (possible use of non-prescribed drugs); Death
15	M	N/A		Salivary hypersecretion; Drug abuse (crack cocaine, marijuana, alcohol); Extrapyramidal disorder	Nonspecific reaction; Treatment noncompliance; Drug abuse (mixed polysubstance abuse); Death
16	F	N/A	Mood stabilizers Antipsychotics		Drug abuse
17	M	1000 mg		Hypercholesterolemia; Substance abuse; Hypertension; Schizophrenia; Gastro-esophageal reflux disease	Hematemesis; Drug abuse; Cardiac arrest; Completed suicide; Toxicity to various agents; Intentional overdose; Resuscitation; Seizure; Tachycardia
18	F, Adult (27yy)	N/A			Drug abuse
19	M, Adult (30yy)	N/A			Myocardial infarction; Drug abuse; Eye swelling; Glossodynia; Tongue coated; Swollen tongue
20	F	N/A			Intentional self-injury; Meningioma; Drug abuse; Hypoglycemia; Peripheral venous disease; Hepatic steatosis; Scar; Pneumonia; Bronchitis; Arteriosclerosis coronary artery; Purulent discharge; Pulmonary congestion; Death
21	M, Adult (26yy)	800 mg			Coronary artery disease; Drug abuse; Hallucination, auditory; Epilepsy; Depressed mood
22	M, Adult (31yy)	N/A			Death; Substance abuse
23	M	N/A		Surgery; Intentional self-injury; Bipolar disorder	Agitation; Death; Drug abuse; Suicidal ideation; Nasal septum deviation
24	M, Adult (30yy)	N/A			Autonomic nervous system imbalance; Catatonia; Drug withdrawal syndrome; Dyskinesia; Neuroleptic malignant syndrome; Psychotic disorder
25	M, Adult (36yy)	N/A			Alcohol abuse; Drug abuse; Myocardial infarction; Coronary artery disease
26	M	200 mg			Drug abuse; Obesity
27	M, Adult (34yy)	350 mg			Circulatory collapse; Intentional product misuse; Cardiopulmonary failure
28	M, Adult (33yy)	N/A	Benzodiazepines Opioids		Death; Drug abuse (heroin, temazepam)
29		N/A		Arm amputation; Drug dependence (including cannabis, benzodiazepines and heroin); Limb reduction defect; Alcohol use	Drug abuse; Loss of consciousness; Hypothermia
30	M, Adult (31yy)	N/A			Abnormal behavior; Myocardial infarction; Drug abuse (heroin, cocaine); Psychomotor hyperactivity; Peripheral coldness; Unresponsive to stimuli; Arteriosclerosis coronary artery; Blood glucose decreased
31.	F, Adult (72yy)	200 mg	Opioids	Schizophrenia; Depression; Somnolence; Anxiety	Movement disorder; Sudden death; Drug abuse
32.	M, Adult (27yy)	400 mg	Antipsychotics	Asthma; Nicotine dependence; Drug abuse; Obesity; Substance use; Diabetes mellitus; Mental disorder; Alcohol use	Asphyxia; Substance abuse
33.	M, Adult (50 yy)	550 mg			Hypoxic-ischemic encephalopathy; Drug abuse (cocaine); Pulmonary hemosiderosis; Pulmonary edema; Cardiac arrest; Left ventricular hypertrophy; Arteriosclerosis coronary artery; Aortic arteriosclerosis; Myocardial fibrosis; Hepatic steatosis; Spleen congestion
34.	M, Adult (29yy)	1000 mg	Opioids	Schizophrenia	Substance abuse (heroin)
35.	F, Adult	N/A	Antidepressants		Drug abuse
36.	M, Adult (34yy)	700 mg		Schizophrenia; Nicotine dependence; Drug abuse	Cardiac arrest; Substance abuse
37.	F, Adult (28yy)	N/A	Antidepressants		Drug abuse
38.	M	100 mg		Blood cholesterol increased; Bipolar disorder; Gastroesophageal reflux; Schizoaffective disorder; Diabetes mellitus	Sudden death; Drug abuse (cocaine and another illicit drug)
39.	F, Adult (50yy)	N/A	Alcohol	Drug abuse; Drug dependence	Completed suicide; Toxicity to various agents; Drug abuse (propofol)
40.	M	N/A		Product use issue; Psychotic disorder	Product use issue; Drug level increased (clozapine levels in the 600,000 s); Death; Adverse event; Psychotic disorder; Intentional product misuse
41.	M	N/A			Drug withdrawal syndrome; Memory impairment; Disorientation; Completed suicide
42.	F, Adult (28yy)	N/A	Antidepressants		Toxicity to various agents; Drug abuse
43.	M, Adult (31yy)	700 mg		Anxiety; Psychotic disorder; Depression	Completed suicide (suicide due to overdose on clozapine); Intentional product misuse; Overdose; Adverse event
44.	M	N/A			Completed suicide (patient jumped to his death); Withdrawal syndrome (periodically he would refrain from taking the medication, thus suffering withdrawal/Initially withdrawal symptoms would consist of disorientation after 2–3 days and memory impairment after 4–7 days)
45.	M	N/A			Death; Withdrawal syndrome (withdrawal symptoms from the capsule form of Clozaril antipsychotic medication)
46.	M, Adult (58yy)	100 mg		Alcohol use; Hypertension	Drug abuse; Vomiting; Asphyxia; Prescription drug used without a prescription (abuse of clozapine 100 mg tablets)

**Table 5:** Reporter’ comments recorded in the line listing; EudraVigilance (EV) clozapine-related misuse/abuse/dependence and withdrawal ADRs (2005-June 2018).

	Comments in the ADRs Reported in the Dataset
**Overdose and suicidal behaviour ADRs**	“Clozapine overdose in suicidal attempt” “Patient stopped treatment and then took an overdose of about 2800 mg” “Clozapine levels in the 600,000’s possible overdose” “Clozapine overdose of 3000 mg/took two weeks supply of Clozaril as 4200 mg (300 mg × 14 dd)” “Clozapine overdose” “Took an overdose of 5600 mg” “The patient took 1300 mg for intentional self-injury” “Suicide due to overdose on clozapine”
**Withdrawal and clozapine discontinuation ADRs**	“Clozaril withdrawal due to non-compliance” “Withdrawal reaction cholinergic rebound” “Anticholinergic withdrawal syndrome” “Withdrawal psychotic episode” “Sudden withdrawal with Clozaril” “Clozaril withdrawal psychosis” “Psychotic decompensation after clozapine discontinuation” “Relapse psychosis” “Experiencing extremely negative reactions due to going off this medication” “Serotonin syndrome associated with clozapine withdrawal” “Withdrawal agitation” “Tachycardia and high BP possibly due to Clozaril withdrawal” “Acute clozapine withdrawal” “Withdrawal symptoms from Clozaril” “Compatible with withdrawal effects arising from the abrupt discontinuation of clozapine”
**Abuse of clozapine**	“Abusing Clozaril taking more than he needs” “Clozaril was prescribed for family members” “Took his wife’s Clozaril to help him sleep” “He depended on clozapine” “Died because of clozapine and alcohol” “Clozapine as a drug of abuse” “Clozaril misuse” “Abuse of Leponex” “Patient possibly has used Leponex as drug of abuse or he bought Leponex for drug abuse by others”
